# A Manual of the Practice of Medicine

**Published:** 1899-09

**Authors:** 


**A Manual of the Practice of Medicine.** By FREDERICK TAYLOR, M.D. Fifth Edition. Pp. xvi., 1002. London: J. & A. Churchill. 1898.

This popular text-book has been carefully revised, some sections have been largely re-written, and notices of many diseases have been added. Amongst the newest chapters are those on glandular fever, divers' paralysis, erythromelalgia, angeio-neurotic œdema, hypertrophic pulmonary osteo-arthropathy, and tubercle of the skin. A separate section is devoted to diseases of the mediastinum; and filarial disease and hæmoglobinuria have been transferred to diseases of the lymphatic system and of the blood respectively. It is difficult to compress the facts of medicine within the limits of a volume such as this, but the issue of five editions in eight years shows that the attempt has been much appreciated.

